# Protocol for a randomised controlled trial of a healthy relationship tool for men who use intimate partner violence (BETTER MAN)

**DOI:** 10.1186/s12889-023-17032-5

**Published:** 2023-12-02

**Authors:** Kelsey Hegarty, Laura Tarzia, Carolina Navarro Medel, Mohajer Hameed, Patty Chondros, Lisa Gold, Simone Tassone, Gene Feder, Cathy Humphreys

**Affiliations:** 1 Department of General Practice & Primary Care, The University of Melbourne, Melbourne, Australia; 2 Centre for Family Violence Prevention, The Royal Women’s Hospital, Melbourne, Australia; 3 School of Public Health, La Trobe University, Melbourne, Australia; 4 School of Health & Social Development, Deakin University, Burwood, Australia; 5 Family Safety Victoria, Melbourne, Australia; 6 Bristol Medical School, University of Bristol, Bristol, UK; 7 School of Social Work, The University of Melbourne, Melbourne, Australia

**Keywords:** Domestic violence, Perpetrators, Digital intervention

## Abstract

**Background:**

Intimate partner violence (IPV) is common globally, but there is a lack of research on how to intervene early with men who might be using IPV. Building on evidence supporting the benefits of online interventions for women victim/survivors, this study aims to test whether a healthy relationship website (BETTER MAN) is effective at improving men’s help seeking, their recognition of behaviours as IPV and their readiness to change their behaviours.

**Methods/design:**

In this two-group, pragmatic randomised controlled trial, men aged 18–50 years residing in Australia who have been in an adult intimate relationship (female, male or non-binary partner) in the past 12 months are eligible. Men who report being worried about their behaviour or have had others express concerns about their behaviour towards a partner in the past 12 months will be randomised with a 1:1 allocation ratio to receive the BETTER MAN website or a comparator website (basic healthy relationships information). The BETTER MAN intervention includes self-directed, interactive reflection activities spread across three modules: Better Relationships, Better Values and Better Communication, with a final “action plan” of strategies and resources. Using an intention to treat approach, the primary analysis will estimate between-group difference in the proportion of men who report undertaking help-seeking behaviours for relationship issues in the last 6 months, at 6 months post-baseline. Analysis of secondary outcomes will estimate between-group differences in: (i) mean score of awareness of behaviours in relationships as abusive immediately post-use of website; (ii) mean score on readiness to change immediately post-use of website and 3 months after baseline; and (iii) cost-effectiveness.

**Discussion:**

This trial will evaluate the effectiveness of an online healthy relationship tool for men who may use IPV. BETTER MAN could be incorporated into practice in community and health settings, providing an evidence-informed website to assist men to seek help to promote healthy relationships and reduce use of IPV.

**Trial registration:**

ACTRN12622000786796 with the Australian New Zealand Clinical Trials Registry: 2 June 2022.

**Version**: 1 (28 September 2023).

## Background

Intimate partner violence (IPV) is a public health problem worldwide. It is defined as any behaviour within an intimate relationship that causes physical, psychological, or sexual harm [1], and is primarily perpetrated by men [2]. In Australia, one in five women report physical or sexual abuse by a male partner, similar to other high-income countries [3]. Men and non-binary people also report male-perpetrated IPV [4, 5]. This violence results in annual costs estimated at $21.7 billion [6], more than well-recognised epidemics such as diabetes [7] and mental health conditions [8]. In addition, children exposed to IPV can experience intergenerational trauma, causing emotional and behavioural problems persisting into adulthood [9]. Thus, there is an urgent need for testing interventions that could help reduce the use of IPV in intimate relationships.

Although IPV can be perpetrated by people of any gender, men use IPV most frequently and severely. Moreover, studies show that IPV is the most common cause of injury for female victims [2, 10]. This has led to IPV research in healthcare settings and online focusing on interventions for women survivors [11, 12]. Yet, current policy and practice suggest that efforts to end IPV must also target men potentially using IPV in their intimate relationships with female, male and non-binary partners [13].

### Responses to men’s use of IPV

Men’s use of IPV is associated with increased alcohol and substance abuse, depression, suicide, anxiety, low self-esteem, and use of health services [14, 15]. Yet responses mainly utilise a criminal justice lens rather than exploring early intervention through health or community settings [16, 17]. In Australia, the preferred referral pathway for men who use IPV is men’s behaviour change group programs (MBCPs). Topics covered by these programs include: masculinity, conflict resolution, anger management, emotional regulation, communication skills, fatherhood, alcohol and drug use, trauma, stress, beliefs that reinforce abuse and maladaptive beliefs [18–20]. MBCPs have mixed evidence of effectiveness [21], with a review of Australian MBCPs suggesting that key issues are getting men to participate and remain in the programs [22]. A systematic review of qualitative studies found that men who volunteer for MBCPs are more likely to sustain change compared with those court mandated or driven by need to be allowed home [23]. Yet only a limited number of MBCP participants enrol in programs voluntarily, whilst more often men are mandated into a MBCP once they enter the justice system. As a result, many men in the community who use IPV in Australia are not reached at an earlier stage in their trajectory of using IPV.

Experts and peak bodies agree that we need to engage men who use IPV earlier to seek help from services [13]. The Commonwealth’s National Plan to Reduce Violence against Women and their Children calls for testing of effectiveness of early intervention models targeting men in the community [13]. To enable this earlier delivery of interventions targeting voluntary men with internal motivations for change, engagement needs to change from justice to health and community settings [24, 25]. However, there is a paucity of evidence from health care settings and no trials of early interventions for men drawn from the community [18]. Theoretically-driven and consumer-informed elements have been successfully transferred from face-to-face to online interventions for other male social problems, such as alcohol misuse [26]. However, evidence of effectiveness for online interventions for men who may be using IPV is still lacking.

### Evidence informing the development and design of BETTER MAN

**Fig. 1:**
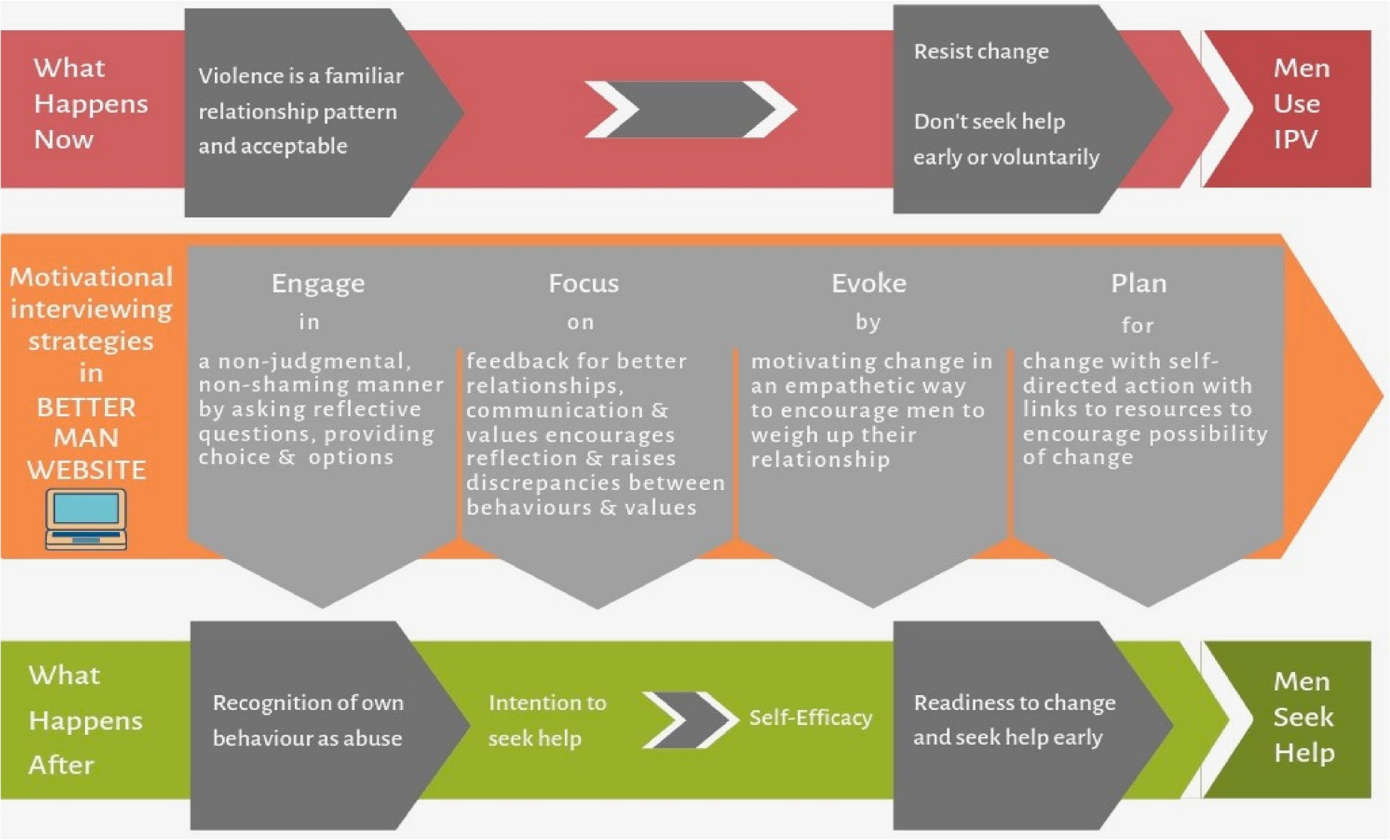
Theory of Change for the BETTER MAN intervention; a pathway to help-seeking to enable safety for partners

To address the gap in knowledge and tools for men who may use IPV, we developed an innovative online early intervention that engages men in the community, providing them with awareness and motivation to seek help for their behaviours before the justice system intervenes. This novel early intervention is an online healthy relationship tool called BETTER MAN. It aims to increase men’s early engagement with help-seeking to ultimately reduce use of IPV (see Fig. 1 for Theory of Change).

BETTER MAN is based on consultation with end-users and sector specialists, guided by research evidence [27], is theoretically-informed [28], and follows best-practice human-computer interaction principles [29].

Development of BETTER MAN involved working with end-users and analysing qualitative data on how to engage men to seek help [27]. In 2017, three focus groups were held with end-users (23 men from MBCPs) who chose the website name, contributed to content and advised on use of appropriate language [27]. In 2018, in two focus groups (21 men from MBCPs) participants suggested using soft language to approach the issue, engaging men through broader health concerns, not making them feel judged and promoting hope for change [30]. In 2019, we undertook a pilot study to test the website content/navigation, ensure usability and confirm project recruitment methods and outcomes [31]. The pilot showed that recruitment to a BETTER MAN trial is feasible, with 162 men enrolling over a three-week period, who then completed online assessments at baseline, immediately on completion of modules (79%, 111 men) and three months post-baseline (63%, 86 men). Further details [31] show the sample included men who were culturally diverse, with 33% born outside Australia (33%). 19% were in a same-sex relationship, and 2.2% identified as Aboriginal and/or Torres Strait Islander. Positive findings included an increase in mean intention to contact a confidential counselling service and a change in men’s readiness to make changes in behaviour. Most men (90%) found the website acceptable, stating they did not feel judged by BETTER MAN. They also stated that it helped them to acknowledge what was happening in their relationship and think about making changes, as well as offering ongoing support. We suggest that BETTER MAN potentially overcomes stigma for men who may be using IPV, as it is seen as private, accessible and non-judgemental, particularly important for men from diverse backgrounds [27, 30]. However, BETTER MAN requires testing through a randomised controlled trial, to enable the website to be rigorously evaluated with a diverse group of men.

## Methods/design

### Aims

The primary aim of the BETTER MAN trial is to determine if an interactive online healthy relationship tool for men in the community who may use IPV results in increased help-seeking behaviours for relationship issues compared to a non-interactive information-only website. The secondary aims are to determine if, compared to a non-interactive information-only website, the intervention:

- increases men’s identification of abusive behaviours in relationships as IPV,
- increases men’s readiness to change their behaviour in relationships,
- is cost-effective.

### Trial design

The BETTER MAN trial is a two parallel group, pragmatic, randomised controlled superiority trial to test an online healthy relationship tool for men in the Australian community who may use IPV and its effectiveness in increasing men’s help seeking behaviours for relationship issues. Individuals will be randomly allocated 1:1 to the intervention or comparison websites. The protocol is described in accordance with the SPIRIT statement [32].

### Participant inclusion criteria

The target population is English-speaking men aged 18–50 years, residing in Australia, who have been in an adult intimate relationship in the past 12 months (female or male or non-binary partner) for longer than one month. Participants need to report concerns about their own behaviour or concerns by others about their behaviour towards a partner in the past 12 months. Additionally, eligible men need to have access to a safe computer or personal device with an internet connection, and be willing to provide their full name, residential address, and email address for validation purposes. A sub-sample of partners of male participants will be invited to take part in a brief survey and interview. Eligibility criteria for partners will be age over 18 years, English-speaking, and with access to a private email account to receive study information.

### Recruitment

Recruitment will be conducted nationally across Australia using a combination of recruitment strategies we have successfully used in previous studies with men who use IPV [31, 33]. The strategies include targeting a wide range of men, not only those who have already acknowledged use of IPV and are using support services. We will recruit men online using a mixture of men’s health, community, or domestic violence-related websites, as well as social media (e.g., Facebook, Gumtree, Google, eBay, Twitter, Instagram). We will also display advertisement material in public spaces such as sports clubs, libraries and hotels. We will reach out to workplaces with large numbers of male employees (e.g., mining and construction companies, unions, banks) to partner with us to advertise the project through newsletters and communications. We will also use online research panels to reach pre-recruited men who suit the trial eligibility requirements. Finally, we will recruit men through people in their close circles (e.g. partners, family members, friends) who feel worried about their behaviour. Online advertisements targeting these people will also be distributed using relevant websites and social media.

Interested men will click directly on the link or scan the QR code provided in the advertisement which directs them to a landing page where information about the project is displayed. Advertisements targeting partners, family members, and friends with concerns about a man’s behaviour in his relationship lead them to a separate landing page with general information about the project and a shareable link to the men’s page. Participants will be able to download a PDF copy of the project information sheet for their records.

From the landing page, men can click through to the study registration website. There, they will be required to complete the eligibility screening questionnaire (age, residence and concerns about behaviour questions) and provide informed consent prior to proceeding. A phone number will be provided for men to contact the research team with queries, as well as a study email address for technical difficulties.

To recruit the sub-sample of participants’ partners, we will follow standard MBCP practice and ask men if they agree to their partners being invited to participate in a brief survey. Those who agree will be asked to provide their partner’s contact details (first name and email address). At the 3-month timepoint, partners will be invited by email to participate in a short online or telephone survey about their relationship (men will not know if their partner participates). Those interested in participating will be directed to a registration website to review the study information and provide informed consent. A phone number will be provided for partners to contact the research team with queries, as well as an email address for technical difficulties. Partners will also be invited to take part in an optional longer interview with a member of the research team.

### Data collection

Study data will be collected and managed using REDCap electronic data capture tools hosted at The University of Melbourne. REDCap (Research Electronic Data Capture) is a secure, web-based software platform designed to support data capture for research studies, providing 1) an interface for validated data capture; 2) audit trails for tracking data manipulation and export procedures; 3) automated export procedures for data downloads to common statistical packages; and 4) procedures for data integration and interoperability with external sources [34, 35].

Table 1 displays the schedule of enrolment, interventions and assessments. In addition to study outcomes (see Table 2) and mediators and moderators (see Table 3), descriptive data collected from men will include socio-demographic information (e.g., age, sexual orientation, education, ethnicity, birthplace, employment, location), relationship data (relationship and co-habitation status, and whether they have children under the age of 18) and self-rated health. Data collected from partners will include basic demographic details, whether they have experienced a significant life event in the previous 3 months, and a simple assessment of relationship health and feelings of safety (using a rating from 0 to 10). Harms and benefits of the study will also be explored through use of the COST questionnaire [36] adapted for perpetrators, and interviews with users and their partners (see Process evaluation section).

**Table 1:**
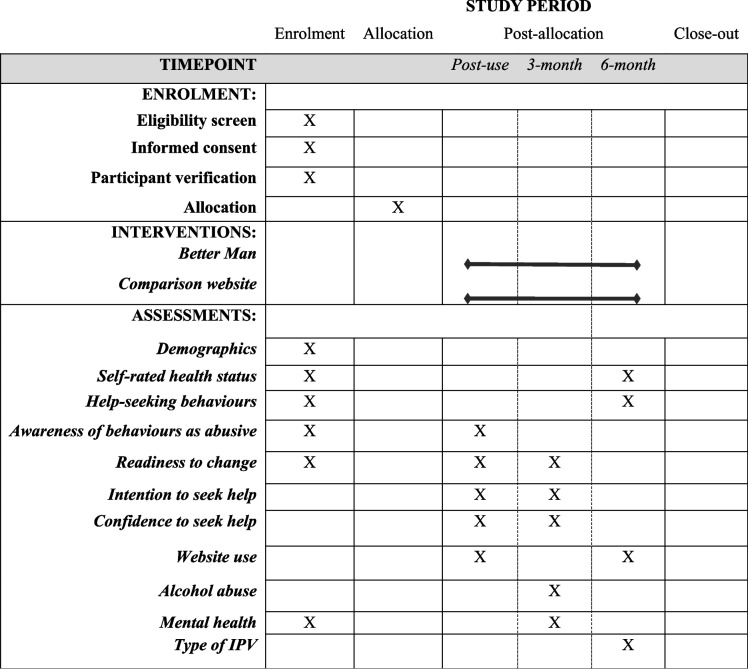
Timepoints, interventions and assessments

**Table 2:** Primary and secondary outcome measures

Outcome (Measure)	Description	Hypothesis
*Primary outcome*		
Help-seeking for relationship issues^1^ at 6 months post-baseline, measured by men’s self-reported contact with counselling services.	Four yes/no questions asking if they have contacted Men’s Referral Service, MensLine, MBCP, or other counselling services for relationships.	A 20% difference in the percentage of men who initiate help-seeking behaviours about relationship issues between intervention (50%) and comparison (30%) groups 6 months post-baseline.
*Secondary outcomes*		
Awareness of behaviours as abusive immediately after intervention completion, measured using the *National Community Attitudes Survey* [37]	Seven questions that ask whether the man regards particular behaviours in relationships as a form of IPV, scored Yes, always; Yes, usually; Yes, some­times; No; Don’t know.	The intervention group will have a higher mean number of items identified as ‘always’ or ‘usually’ IPV compared to the comparison group, immediately after intervention completion.
Readiness to change measured using a modified version of the *Contemplation Ladder* [35, 36] immediately after website use and 3 months post-baseline.	A single question that measures how ready a man is to make positive changes to his behaviour by selecting one of 10 statements that best reflect his feelings. Range of values will be between 1 and 10, with a higher score indicative of greater readiness to change.	The intervention group will have a greater readiness to change mean score than the comparison group, immediately after intervention completion and 3 months post-baseline.
Costs presented in Australian dollars. Additional cost per additional man initiating help-seeking	Cost assessed after 6 months post baseline as the additional cost per additional man initiating help-seeking. Costs of managing and operating the intervention and comparison websites will be calculated from study records. Men’s time costs will be measured via recorded website analytics (time spent on website) and valued at the average Australian wage rate. Costs will also include time of stakeholders and cost of services used.	The intervention is cost-effective, compared to a comparison website measured at 6 months post-baseline.

^1^These questions were developed for the purposes of this study

**Table 3:** Mediators and moderators

Variable (measure)	Description
*Mediators and moderators*	
Perceived intention to seek help for relationship issues (visual analogue scale^a^)	A single 1–10 rating question of intention to contact helplines (e.g., Men’s Referral Service, MensLine), MBCPs, or other counselling services for relationships. A higher score indicates stronger intention to seek help.
Perceived self-efficacy to seek help for relationship issues (visual analogue scale^a^)	A single 1–10 rating question of confidence in ability to contact helplines (e.g., Men’s Referral Service, MensLine), MBCPs and other counselling services for relationships. A higher score indicates higher self-efficacy.
Website use (Time and page access of the website^a^) [38]	Software will record number of times the intervention website is accessed by each man, the time spent on the intervention website each time, and number of pages visited.
Alcohol abuse (*Alcohol Use Disorders Identification Test, AUDIT* [39]	A 10-item screening tool developed by the World Health Organization to assess alcohol consumption, drinking behaviours, and alcohol-related problems. Responses are scored from 0 to 4, giving a maximum possible score of 40. A score ≥ 8 is associated with harmful or hazardous drinking; a score ≥ 15 or more is likely to indicate alcohol dependence in men.
Depressive symptoms (*The Patient Health Questionnaire-9, PHQ-9* [40]	A self-administered version of the depression module in the PRIME-MD diagnostic instrument for common mental disorders. It assesses the presence of 9 DSV-IV symptoms of depression over the past 2 weeks, scored on a 4-point Likert Scale from “0” (not at all) to “3” (nearly every day). Sum of the 9 items ranges between 0–27, with higher scores indicating more severe depressive symptoms.
Depressive symptoms (*The Patient Health Questionnaire-2, PHQ-2* [41]	Two-item version of the PHQ-9 [41], inquiring about the frequency of depressed mood and anhedonia over the past two weeks. Item responses range from 0 (Not at all) to 3 (Nearly every day). The total score ranges from 0 to 6.
Post traumatic stress symptoms (*Short screening scale for DSM-IV posttraumatic stress disorder* [42]	A seven-symptom screening scale for PTSD. Total score ranges from 0 to 7. A score ≥ 4 defines positive cases of PTSD.
Anxiety symptoms (*The Generalized Anxiety Disorder scale-2, GAD-2* [43]	A 2-item version of the GAD-7 anxiety scale that asks about the core anxiety symptoms. Sum of the scores range from 0 to 3, with higher scores indicating more severe anxiety symptoms.
Type of IPV (*The Composite Abuse Scale- Short Form, CASR-SF* [44]	Fifteen Yes/No items that capture physical, sexual and psychological abuse and overall IPV. If Yes then a frequency scale ranging from “not in the past 12 months” (0) to “daily/almost daily” (5) is presented. Total scores range from 0 to 75, representing the mean of past 12-month abuse experiences responses multiplied by 15.

^a^These questions were developed for the purposes of this study

### Trial process

**Fig. 2:**
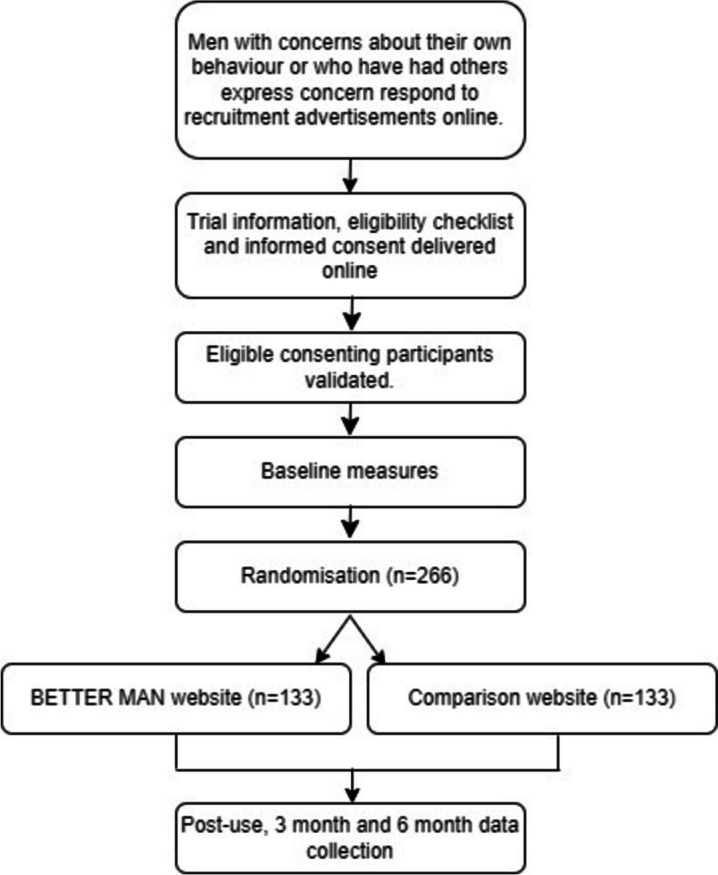
Flow of participants through trial

 The flow of participants through the trial is illustrated in Fig. 2. A man who has met initial eligibility criteria in the screening survey will be asked to provide informed consent and personal details (full name, residential street and suburb, email address). These details will be manually checked against the Australian Electoral Roll by a team member as a safeguard against fraudulent participants and bots. If the person’s details are not on the Electoral Roll, the participant will be contacted by email and asked for further information. If there is a valid reason for a participant not being registered to vote (e.g., due to age or visa status), he will be asked to confirm his details with a research assistant over the phone. Once the participant’s identity is validated, he will be accrued into the study. Upon verification, participants will receive an automated email with a personalised link to the baseline survey; the link will be deactivated once the participant has completed the survey.

Participants will have a 30-day window in which to log in to complete their baseline visit. Automated reminder emails will be sent at regular intervals throughout the 30-day window until the baseline measures are completed. An enrolled participant who has not completed baseline measures in the allocated timeframe will be deemed off the trial and not randomised.

After completion of the baseline measures, men will be randomised into either the intervention or comparison group. They will be required to complete another short survey of up to five minutes immediately post-use of the allocated website. For men allocated to the intervention, the overall time commitment could be up to 60 min, and for the comparison group up to 30 min. Men will not be required to complete their visit all in one sitting but can log out and back in at another time.

At 3 and 6-months post baseline, participants will be sent emails to invite them to complete follow-up surveys. It is expected that 3 and 6-month surveys will take up to eight minutes. The actual time spent by men either in the intervention or comparison website will be recorded by the website analytics, and this will be used for both process evaluation and to inform cost-effectiveness.

Eligible partners who provide consent will receive an email with a link to the survey. Those who prefer a telephone survey will be called by one of the researchers and will answer the 10-minute survey questions about how safe they feel. Three months later, partner participants will be sent emails to invite them to complete the survey again either online or by phone.

### Randomisation and blinding

Participants will be randomly assigned with a 1:1 allocation ratio to the two-study groups using a computer-generated random allocation schedule. The schedule will utilise block randomisation with permuted blocks of random sizes to ensure the number of individuals are balanced across the study groups. To ensure concealment, the block sizes will not be revealed until recruitment is completed. The random allocation schedule will be uploaded into the REDCap randomisation module [35] by the statistician. After participants complete the baseline measures, they will automatically be randomly allocated and sent a link to either the intervention or comparison website. They will not be informed as to whether the website they have been assigned to is the intervention or comparison site, although it is possible that they may guess which website they are using. A list of participants’ names and study group allocations will be stored separately to ID numbers and contact details within the secure study database. One team member, who will monitor the randomisation process, will be unblinded to the participants’ allocation. All other BETTER MAN investigators and research team involved in analyses will be masked to the group allocation of participants until after collection and analysis of the 6-month data. Until this time, the two groups will be referred to as Group A and Group B (with the intervention group randomly allocated to either letter by the website developer/administrator). Follow-up for those who fail to log on and complete baseline, 3 or 6-month measures will be done by a research assistant not connected with any of the data management or analyses.

### Retention

Several strategies will be used in order to maximise retention in the study. Given that the trial will be conducted entirely online, face-to-face contact with a research assistant will be non-existent or minimal. It is therefore essential to maintain contact with men in other ways, including:

- Regular emails to ‘touch base’ and remind participants about the study.
- Gift certificates to show appreciation for men taking the time to participate, at immediate post-use and at 3 and 6-month follow-up (up to a maximum of $120 per participant across the study duration: $30 at immediate post-use, $40 at 3 months and $50 at 6 months). Vouchers will be emailed to their nominated email address with an accompanying email thanking them for their participation.
- Use of friendly, warm communication strategies in all communication to enhance the feeling of support.

Similar strategies will be implemented for ensuring retention of partner participants, including:

- Gift certificates to show appreciation for partners taking the time to participate, at 3 and 6-month follow-up (e.g., $30 at 3 months and $30 at 6 months).

### Intervention group (the BETTER MAN website)

BETTER MAN is a healthy relationship website for early intervention that offers a private, accessible and non-judgemental resource for men who may use IPV. It applies behaviour change principles to promote men’s readiness to change using the well validated Transtheoretical Model psychological theory on which motivational interviewing (MI) is based. MI is a person-centred, strengths-based technique to enhance motivation to change through challenging ambivalence to increase help-seeking [45]. Previous MI approaches to engage men prior to MBCPs have demonstrated significant increases in program attendance and completion [20, 46]. MI techniques [45] used throughout BETTER MAN encourage awareness and self-reflection, reduce stigma, and improve communication skills. They enable men, who will vary in their readiness to change, to weigh up their situation and options, to form action plans that meet their personal priorities, tailored to their self-perceived Stage of Change (pre-contemplation, contemplation, preparation, action and maintenance). With the ultimate aim of improving safety for partners and children, BETTER MAN involves the following MI techniques: Engage, Focus, Evoke, Plan (see Fig. 1).

The BETTER MAN website is composed of three sequential modules: Better Communication, Better Values, and Better Relationships, with all modules having online messaging tailored to user input. The Better Communication module explores how a man’s communication style may differ with a partner compared to others, and promotes positive communication (e.g., listening, staying calm). The module also contains a grounding breathing exercise and an activity for recognising signals of potentially abusive behaviour. The Better Values module explores how a man’s behaviour in relationships aligns with his self-identified values using hypothetical scenarios to highlight discrepancy between values and behaviour. The Better Relationships module encourages a man to reflect on the health of his relationship and his use of controlling behaviour and violence (physical, emotional, sexual abuse), and weigh up pros/cons of his relationship (including with his children if applicable). A final Action Plan section reinforces help seeking and provides resources for related areas (e.g., parenting, alcohol/drug use, mental health). The modules take about 30 min and men can go back to the website any time.

### Comparison group

Men in the comparison group will be directed to an alternate website representing the usual available resources and standard care for men seeking help online in Australia. This website will be specially built for the study based on content from HealthDirect, a government-funded service providing quality, approved information about a range of health topics. One of the website sections is about how to build and maintain healthy relationships with partners and family members. The section answers questions such as: What are the signs of a healthy relationship? What are the benefits of healthy relationships? Communication in a healthy relationship? How can I maintain healthy relationships? The website also provides a list of resources for support, a comprehensive directory of Australian websites that people can search for further related information and a 24-hour health advice hotline. The comparison website will be built in a way that reflects the contents in the HealthDirect section on healthy relationships.

### Ethics, safety and data monitoring

Ethics approval for this study was obtained from the Human Ethics Research Committee at the University of Melbourne (HREC 1,442,953).

#### Safety of participants and researchers

Information is provided to all participants (men and partners) regarding the safe use of computers and the internet. Additionally, BETTER MAN is designed with a ‘quick escape’ bar that allows immediate exit and log out from BETTER MAN and returns the user to a weather forecast website. All automated emails sent to participants have the subject header “Better Man Project” and come from a generic email address (mens-health@unimelb.edu.au). Should a participant contact a member of the research team because they are upset due to their participation in BETTER MAN, the researchers will refer to the study distress protocol in their response. The protocol includes exploring with the individual whether they have someone they can talk to who will understand and be supportive. The research team member will discuss with the participant where they might seek support, including from the agencies detailed on the resource list provided to all participants. In the unlikely event that a participant discloses child sexual abuse in their answer to the few open-ended questions in the surveys, researchers will follow State-based mandatory reporting requirements. Participants are able to withdraw from the trial at any time on request.

Regular team meetings will be held for researchers to debrief about the project and any incidents that have arisen. If researchers are distressed at any time from the work, they will be offered referrals for appropriate counselling.

#### Data storage, monitoring and dissemination

Deidentified datasets downloaded from REDCap will be stored on password-protected computers at The University of Melbourne, with access limited to research team members named on the ethics application. Deidentified data will be retained for a minimum of five years after study completion.

An independent Data Monitoring Committee (DMC) has been established to ensure that the trial is conducted appropriately and safely. The DMC will meet at least once a year, following data collection milestones or major trial events. The DMC is composed of diverse experts in randomised controlled trials and IPV. They will ensure that trial participants are protected and will monitor the overall conduct of the trial. To further enhance adherence to study processes, all staff members involved in the trial will have access to a detailed protocol document, as well as the approved ethics application.

The study findings will be disseminated through peer-reviewed publications, presentations at academic conferences and for the sector, and via social media and other online platforms. No identifying details will be used when reporting study findings.

### Outcomes

Outcomes, measures, and hypotheses for the BETTER MAN trial are shown in Table 2. Primary outcome is difference between the intervention and comparison groups in the percentage of men who report undertaking help-seeking behaviours for relationship issues in the last six months (as measured by men’s self-reported use of MensLine, Men’s Referral Service, MBCP, or other counselling services for relationships), 6 months post-intervention. The secondary outcomes are differences between the two study groups in:

1. mean number of behaviours in relationships identified as abusive as measured by the 7-item “Understanding of Violence against Women Scale” from *The National Community Attitudes towards Violence against Women Survey* [37], immediately after website use;
2. mean score on readiness to change as measured by 10 items adapted from the *Contemplation Ladder* [47, 48], immediately after website use and 3 months post-baseline.
3. cost-effectiveness at 6 months post-baseline.

Additional data will be collected as hypothesised mediators and moderators (see Table 3). Mediators of the outcomes include intention and confidence to seek help (rating scale 1–10), alcohol use, depression symptom severity and use of the website (accessed, time spent, pages visited in 6 months). Moderators of the outcomes include baseline levels of mental health (depressive, anxiety and PTSD symptoms) and type of IPV perpetrated.

### Sample size

Based on the response rate (108/137, 79%) from the BETTER MAN pilot [31] and allowing for attrition rate of 30% at six months (based on previous studies [39]), 266 men at baseline (133 per group) will provide 80% power (alpha 5%, 2-sided test) to detect a meaningful difference of 20% (based on our pilot work [31]) in the percentage of men who initiate help-seeking for relationship issues between intervention (50%) and comparison (30%) groups. Additionally, based on the pilot, we expect up to 15% of partners will be recruited.

### Data analysis

#### Statistical methods for primary and secondary outcomes

Participants’ characteristics will be summarised using means and standard deviations (or percentiles as appropriate) for continuous data, and frequencies and percentages for categorical data, for both study groups. Primary analysis will use an intention to treat approach [49], where all individuals will be included in the analysis according to the study group they were assigned, whether they completed all, part or none of the intended intervention.

Logistic regression will be used to estimate the odds ratio of men’s help seeking activities at 6 months for the intervention against the comparison group. Generalised linear model with the identity link function and binomial family will be used to estimate the absolute difference in the percentage of men’s help seeking activities between the two study groups. Both models will adjust for whether the participant had children or did not have children and self-reported help-seeking behaviours measured at baseline. The estimated intervention effect for the primary outcome will be presented as both an odds ratio comparing the intervention to the comparison group (relative effect) and between-group difference in the percentages (absolute effect) with their respective 95% confidence intervals (CI), and the p-values estimated using logistic regression.

Mixed effects linear regression models will be used to estimate the between group difference in the mean scores for the secondary continuous outcomes, namely, awareness of behaviours as abusive immediately after the use of the website completion, and readiness to change at two assessment time points, after the use of the website completion and 3 months post-baseline. Study group and time of measurement and whether the participants had children will be included as fixed effect and random intercept terms for individuals (to account for repeated measurement). A two-way interaction between the study group and time will be fitted to estimate the between-group difference in mean outcome at each measurement time point, but we will constrain the baseline means between the study groups to be equal. The estimates will be presented as difference in the mean outcome between the intervention and comparison groups at immediate post-intervention and 3 months (as appropriate), with 95% confidence intervals and p-values.

Handling of missing data will be detailed in a statistical analysis plan (SAP) informed by a blinded review of data. The SAP will also elaborate on additional analyses such as sensitivity analyses, analysis to assess non-adherence to the intervention on the estimated intervention effects and mediator/moderator analyses. The SAP will be made available prior to the statistical analysis of the primary outcome.

### Economic evaluation

For the economic evaluation, resource use data from website software and men’s self-report will be valued at standard unit cost rates to estimate intervention delivery costs and impact of the intervention on costs of access/use and service use over the six months from the perspective of (i) government and (ii) men. The six-month time duration avoids the need for discounting. Economic evaluation will, as a first step, present a cost-consequences analysis with costs presented separately for government and men compared with the difference in all outcome measures. Cost-effectiveness will then be presented on the primary outcome, in terms of the additional cost per additional man reporting help seeking at 6 months.

Statistical analyses will be conducted in Stata Statistical software 17 [50].

#### Process evaluation

Process evaluation will focus on men’s experiences of behaviour change over time and the potential role of the BETTER MAN website in this process. We will use Interpretative Phenomenological Analysis methods [51] to explore the meanings men attach to behaviour change and how these meanings change over time. We will also examine what supports or services have been helpful and what factors may impede or facilitate change processes. We will also ask men to reflect on what elements of the BETTER MAN website may have been useful to them and why.

Semi-structured, in-depth interviews will be conducted via telephone with a sub-sample of up to 30 men and their partners (we estimate up to 10 partners may be recruited based on our pilot work) immediately after completion of BETTER MAN, and at 3 and 6-months post-use. We will recruit by asking men who complete the post-use survey if they are interested in taking part in an interview. Where possible, we will aim for diversity in terms of demographic factors (e.g., cultural background, location, sexual orientation). All interviews will be recorded and transcribed verbatim. Our analysis will take a trajectory approach [52], which aims to examine the experiences of the same group of participants over time. Analysis will first focus on the individual level, before examining patterns of change across the cohort.

## Discussion

Evidence to inform practice and policy about early intervention with men’s use of IPV in community and health settings is urgently needed. There is a strong rationale for developing and testing online early interventions to reach men in the community who may use IPV. With an increasing demand for services and a community sector that is overworked, the community needs novel ways of addressing the issue of IPV that are rigorously evaluated. This trial evaluates primarily the effect of an online healthy relationship tool on men’s help-seeking for their behaviours in intimate relationships. Well-designed theoretically and consumer informed interventions need testing through randomised controlled trials that incorporate economic and process evaluations. If successful, the trial would provide strong evidence for an entirely new way of addressing and assisting men who might use IPV to seek help. With evidence for effectiveness and cost-effectiveness, the evidence-informed website to assist men to seek help to reduce use of IPV, could be easily incorporated into practice both within the community sector and in health settings. BETTER MAN also has the potential to be scaled up for international delivery. The trial will also add to the knowledge base around internet-based trials, online recruitment of men and retention processes.

### Trial status

Currently recruiting.

## Data Availability

Not applicable.
